# Supplementary material for: Photonic Majorana quantum cascade laser with polarization-winding emission
Source: Nat Commun. 2023 Feb 9;14:707. doi: 10.1038/s41467-023-36418-y (PMC9911720; doi:10.1038/s41467-023-36418-y)
Supplement: Supplementary file 1 — Supplementary Information [file 41467_2023_36418_MOESM1_ESM.pdf]

Supplementary materials for "Photonic Majorana Quantum Cascade Laser with Polarization-Winding Emission"

Song Han[1, †], Yunda Chua[1, †], Yongquan Zeng[2, *], Bofeng Zhu[1, 3], Chongwu Wang[1], Bo Qiang[1], Yuhao Jin[1], Qian Wang[4], Lianhe Li[5], Alexander Giles Davies[5], Edmund Harold Linfield[5], Yidong Chong[3], Baile Zhang[3], Qi Jie Wang[1, 3, *]

[1]Centre for OptoElectronics and Biophotonics, School of Electrical and Electronic Engineering & The Photonics Institute, Nanyang Technological University, Singapore, Singapore.

[2]Electronic Information School, Wuhan University, Wuhan, China.

[3]Division of Physics and Applied Physics, School of Physical and Mathematical Sciences, Nanyang Technological University, Singapore, Singapore.

[4]Institute of Materials Research and Engineering, Agency for Science, Technology and Research (A*STAR), 2 Fusionopolis Way, #08-03, Innovis, Singapore 138634, Singapore.

[5]School of Electronic and Electrical Engineering, University of Leeds, Leeds, UK.

[†]These authors contributed equally to this work

*Corresponding author: yqzeng@whu.edu.cn; qjwang@ntu.edu.sg

1. Emission characteristics of conventional ridge lasers

To investigate the gain spectral range of the THz QCL wafer, conventional ridge laser has been fabricated and characterized. **Figure S1a** plots the emission spectra of a ridge laser at pump current densities scanning through the entire dynamic range. The gain spectral range is approximately from 2.9 THz to 3.9 THz. With increasing pump, the emission spectrum envelope gradually blueshifts, which is due to the Stark shift of the intersubband transition in the THz quantum cascade medium [1, 2]. These results also helped us to estimate the effective refractive index (based on the FSR) of the QCL active region to be around 3.85 at the operation frequency. To visualize the active region design information, the intersubband transition process is plotted in **Figure S1b**, where the electron wavefunctions were calculated by the tight-binding model. The active region consisted of 228 module repeats with alternating barriers ($Al_{0.15}Ga_{0.85}As$) and quantum wells (GaAs). Starting from leftmost barrier of each period, the layer thicknesses in nm (with barriers indicated in bold-face font) are 8.15/**2.46**/8.9/**4.3**/16/**4.1** and the widest well is n-doped with Si at 7×10^{16} cm^{-3} in the central 5 nm region. The bias voltage is 59 mV/module. The corresponding gain profile for the QCL with one period of 44 nm in thickness is also calculated, as shown in **Figure S1c**. The applied electric field is 59 mV that corresponds to 13.4 V if the overall thickness of the real QCL is 10 μm [2]. This is lower than the measured devices that is reasonable as the fabricated devices have contact Ohmic loss, the grown Au films above/below the QCL exist impurities, and the conduction wires inside the cryostat exist Ohmic loss, and so on. This estimated gain is therefore employed to match the radiative losses in the main text for calculating net gains of each photonic mode.

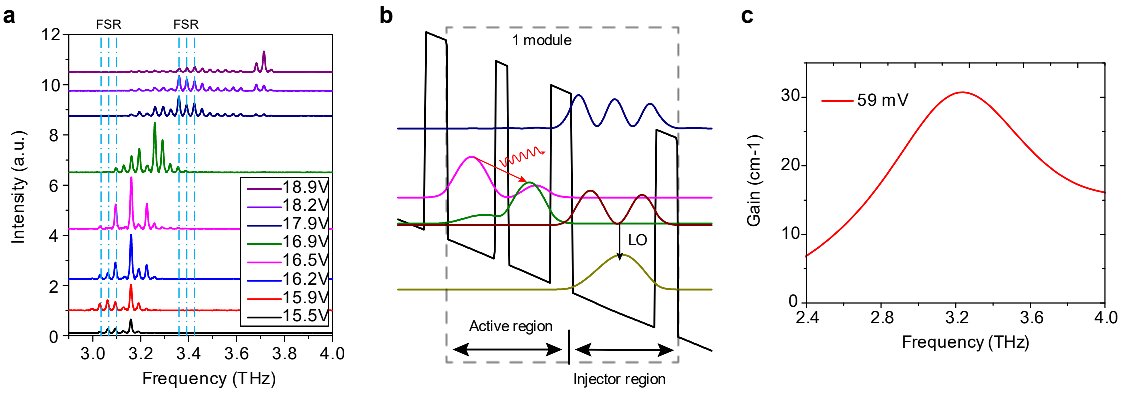

Figure S1. **a**, Emission characteristics of a conventional ridge laser with length of 1500 μm and width of 100 μm fabricated on the quantum cascade wafer. **b**, active region design with one module of 44 nm in thickness. **c**, Calculated gain profile for the QCL at pump voltage of 59 mV.

2. THE JACKIW-ROSSI MODEL

Our system consists of a hexagonal superlattice of air cylinders drilled in an otherwise isotropic QCL slab. Recall that a graphene-like tight-binding model reduces to a two-valley Dirac Hamiltonian close to the Dirac point [3]:

$$H = \begin{bmatrix} 0 & t_1 & 0 & t_2 e^{i\vec{k}\cdot\vec{a}_1} & 0 & t_1 \\ t_1 & 0 & t_1 & 0 & t_2 e^{i\vec{k}\cdot\vec{a}_2} & 0 \\ 0 & t_1 & 0 & t_1 & 0 & t_2 e^{i\vec{k}\cdot\vec{a}_3} \\ t_2 e^{-i\vec{k}\cdot\vec{a}_1} & 0 & t_1 & 0 & t_1 & 0 \\ 0 & t_2 e^{-i\vec{k}\cdot\vec{a}_2} & 0 & t_1 & 0 & t_1 \\ t_1 & 0 & t_2 e^{-i\vec{k}\cdot\vec{a}_3} & 0 & t_1 & 0 \end{bmatrix}. \quad \text{(S1)}$$

where t_1 and t_2 represent the intra- and intercell interactions between the neighboring lattice sites, respectively, $\vec{k}$ is the in-plane wave vector; $\vec{a}_1$, $\vec{a}_2$, and $\vec{a}_3$ are the three lattice vectors with $\vec{a}_3 = \vec{a}_2 - \vec{a}_1$. **Figure S2** shows the bulk band structures calculated from Eq. S1. For the hoppings $t_1 = t_2$, the cylinders create a honeycomb channel for wave propagation, which leads to the formation of two branches (valleys) of Dirac-cone dispersive states outside of the unperturbed band. However, for $t_1 \neq t_2$, the intervalley coupling is realized by introducing a finite mass term, which opens a bandgap at the Γ point. Specifically, for the inter-supercell hopping is larger than the intra-supercell hopping ($t_1 > t_2$), the system shows topological trivial property, while for the inter-supercell hopping is smaller than the intra-supercell hopping ($t_1 < t_2$), a band inversion is overserved that characterizes a topological phase transition.

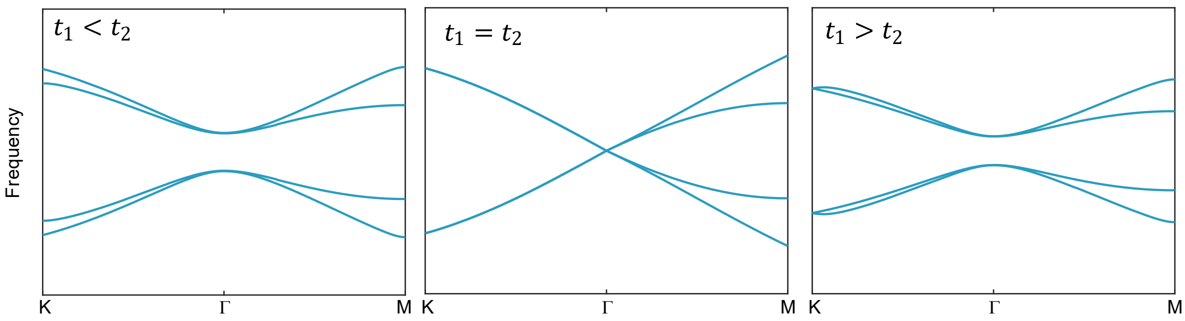

Figure S2. Band structure of the photonic lattice with different relative hopping amplitudes of t_1 and t_2.

We now consider the intervalley coupling by breaking the symmetry with fixed modulation phase. This needs a modulation in the radius of the air cylinders drilled in the QCL slab. Periodic boundary condition is therefore applied to the unit cell structures, and the corresponding radius at each site under modification is given as

$$R(\boldsymbol{r}) = R_0 + \Delta R(r)\cos[\boldsymbol{K}\cdot\boldsymbol{r} + \theta]. \quad \text{(S2)}$$

Here $r = (0,0)$, $(a/\sqrt{3},0)$, $(a/2\sqrt{3},a/2)$, $(-a/2\sqrt{3},a/2)$, $(-a/\sqrt{3},0)$, $(-a/2\sqrt{3},-a/2)$, and $(a/2\sqrt{3},-a/2)$. The variation of cylinder radius modifies the hopping amplitude $t_{1,2}$ in the low-energy model by a certain $\delta t(r)$. The form of R(r) thus modulates the (real) hoppings as $t(r) = t_0 - \delta t(\boldsymbol{r})\cos[\boldsymbol{K}\cdot\boldsymbol{r} + \theta] = t_0 + \left[\Delta(\boldsymbol{r})e^{i\boldsymbol{K}\cdot\boldsymbol{r}} + \Delta(\boldsymbol{r})e^{-i\boldsymbol{K}\cdot\boldsymbol{r}}\right]$, where $t_0 = t_{1,2}$, and $\Delta(\boldsymbol{r}) = -\frac{1}{2}\delta(r)e^{i\theta}$ is the band opening. Therefore, the band gap opening due to intervalley coupling is mainly controlled by the modulation phase θ, as shown in **Figure S3**. Interestingly, the supercell corresponds to the topological phase transition ($t_1 < t_2$) in **Figure S2** for a given modulation phase θ = 0°, and it corresponds to trivial phase ($t_1 > t_2$) in **Figure S2** for a given modulation phase θ = 180°, respectively.

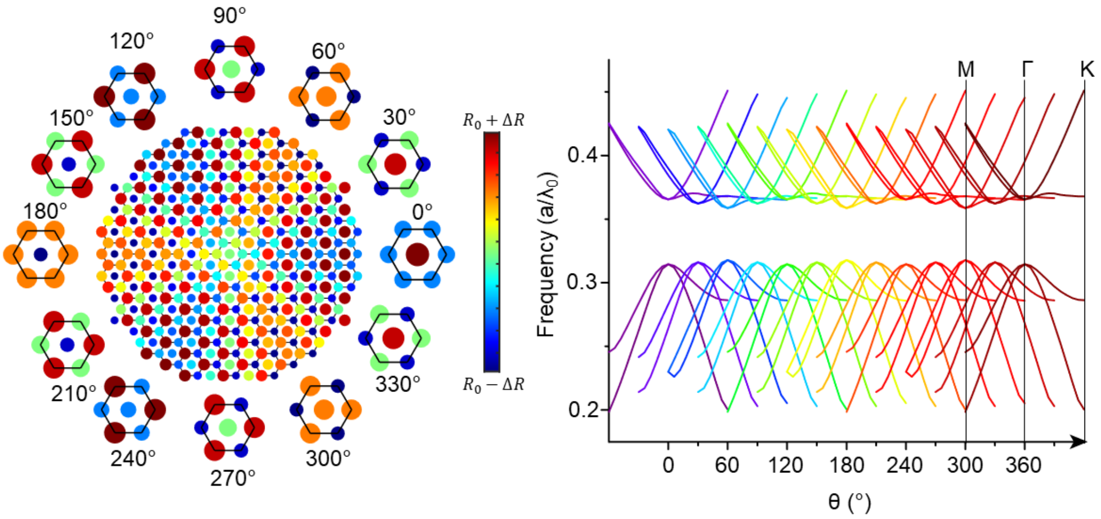

Figure S3. Bulk band structures at fixed phases from 0 to 2π with a step of π/6.

3. Temperature-dependent laser performance and robust far-field beam profiles

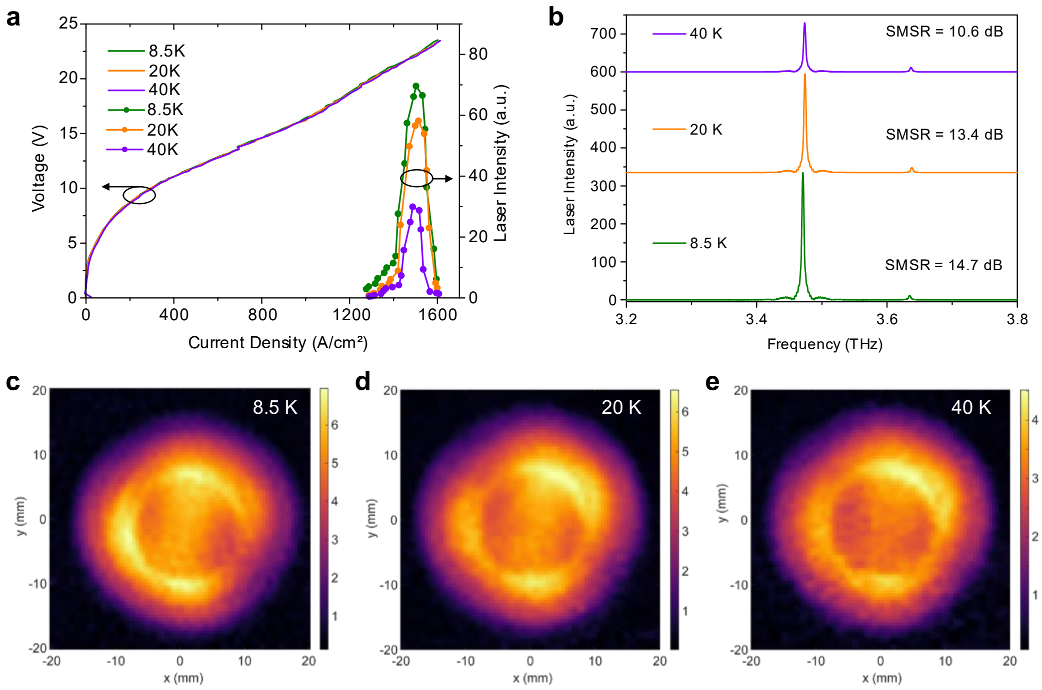

Figure S4. Temperature-dependent performance of the photonic Majorana-like single-mode QCL. a, the L-I-V curves at cooling temperatures of 8.5 K, 20 K, and 40 K, respectively. **b**, the corresponding lasing spectra at their maximum output. **c-e**, the measured far-field beams show that they maintain consistent profiles even the SMSR deceases due to temperature rising.

To investigate the lasing spectra and far-field emissions at different temperatures, we measured the topological QCL device with +1 winding number, accordingly. **Figure S4a** shows the L-I-V curves at three temperatures, i.e., 8.5 K, 20 K and 40 K, respectively. The I-V curves share same trends when the external pump increases. This means that the resistivity of the QCL stays almost unchanged even the temperature varies. However, the light intensities decrease proportionally, which indicates the phonon absorption gets stronger as temperature increases. The SMSR also decreases from 14.2 dB for 8.5 K to 10.6 dB for 40 K but the Majorana lasing mode is always dominant and pined at invariant lasing frequency during such process, as shown in **Figure S4b**. Most importantly, the emitted beams are measured as it has been illustrated that they must show CVB profiles, as shown in **Figures S4c-S4e**. We observe the robust laser profiles, showing donut shapes, even the SMSR decreases due to temperature rising. Such phenomenon, in some sense, demonstrates the topological robustness of the Majorana-like photonic QCLs that not only for the lasing frequency, but also for the emitted far-field beam profiles.

4. High-performance THz topological MZM QCL at large scale

To further estimate the high-power emission with single-mode operation, we calculated the large-scale devices at different vortex core, while the width of the Kekule part is kept in same, as shown in Figure S5a. As the core radius increase, the photonic Majorana zero mode (MZM) firstly red-shifts and then pins around 3.4 THz. In addition, the Q-factor of the MZM is also largest. Although some additional modes will enter the photonic bandgap, the free-spectra range (FSR) is relatively large to allow single-mode lasing at large-area device. With a high-power QCL wafer and large-area design, e.g., vortex core radius $\xi = 32a$ or even at $\xi = 64a$, the Q-factor and pump area of the MZM cavity are significantly enhanced for high-power emission. Experimentally, a sample with vortex core radius $\xi = 8a$ was fabricated and measured. As shown in Figure S5c, the measured far field profile has a good match to the theoretical calculation (Figure S5b), except for a slight beam distortion due to the alignment inaccuracy. In a word, here we show that the emission power of topological MZM QCL can be further improved by simply scaling the cavity size while maintaining single-mode operation and CVB emission with nontrivial polarization winding.

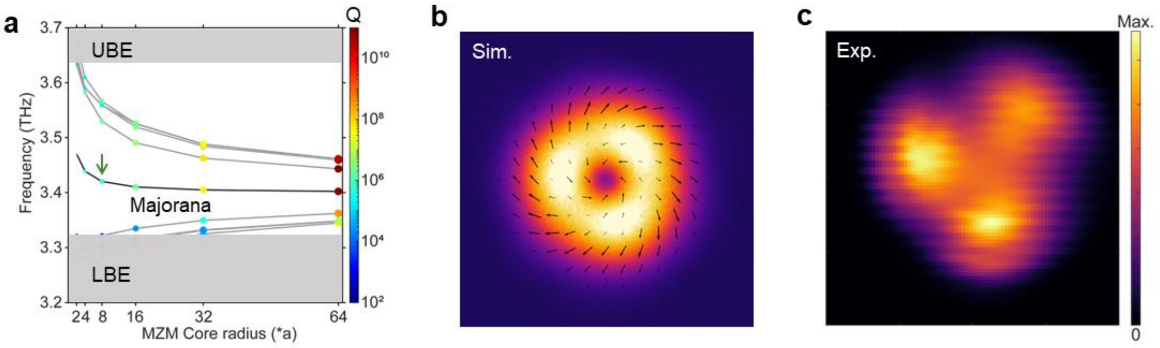

Figure S5. a, 2D simulation for the devices at different MZM core radius (ξ). The numerical simulated (b) and experimentally measured (c) far-field beam profiles for the device with ξ = 8a.

5. Fit the expansion the cylindrical air holes after RIE and acid etching

To fabricate the samples, the UV lithography and the dry etching process was used to drill air holes through the III-V semiconductor materials. After the dry etching, it's clearly observed that the side walls of the air cylinders exist residuals. To remove these residuals, the final devices were cleaned by a mixture of $H_2SO_4/H_20_2/H_20$ with the ratio of 1/8/80. It was found that the etching depth, size expansion, and undercut were closely related to the air hole radius. For air hole with smaller radius, the etching rate is smaller due to the experience that a larger aspect ratio slows down the removal of etching byproducts and the replenishment of etching gases and acid. Therefore, the smaller air holes are featured with slightly less etching depth, smaller size expansion and etching undercut, as shown in **Figure S6**. It was also found that the air holes were not well patterned. The exact shape is slightly distorted to be elliptical with ratio of long axis divided by short axis as 1.05, and orientation along y-axis, which leads to slight lattice anisotropy, as shown in **Figure S7a**. In addition, the microscopic image of a real fabricated device was analysed to estimate the averaged radius variation $\Delta R = \Delta R + \delta R = (1+k)\Delta R + \delta R_0$, which can be fitted by a linear function with slope $k = 0.0203$ and $\delta R_0 = 0.1635$ μm, as shown in **Figure S7b**. Taking the linear expansion of air holes, lattice anisotropy, as well as realistic metal losses into account, all the eigen-modes are calculated by the Finite-element method.

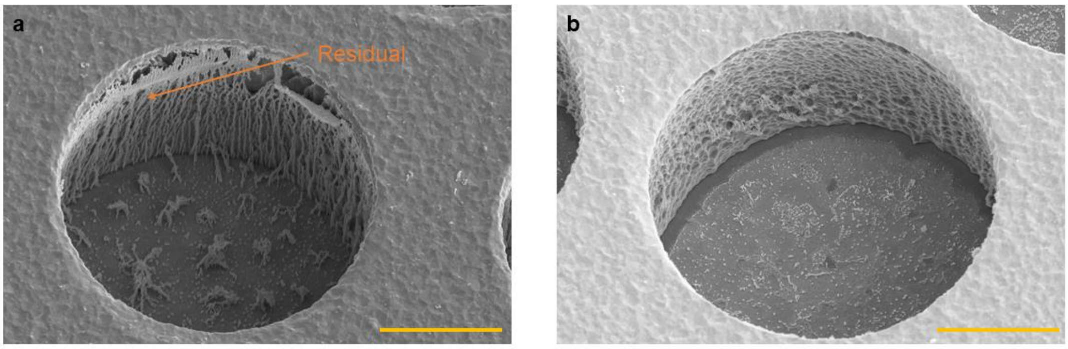

Figure S6. The fabricated samples **a**, before and **b**, after the acid etching. Scale bar indicates 5 μm.

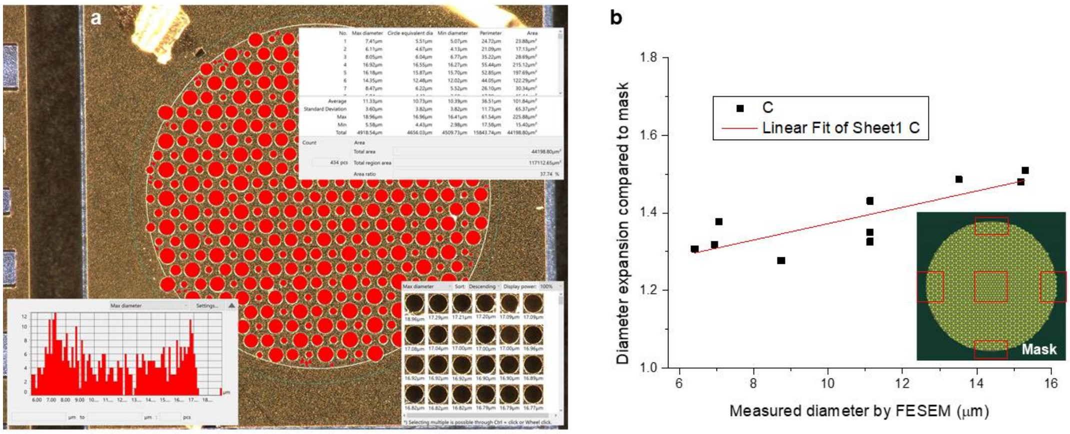

Figure S7. **a**, Microscopic image captured by the Keyence digital microscope (model: VHX-7000 series). The ratio of semi-major axis over semi-minor axis is given by the averaged maximum diameter over the minimum diameter that is directly given by the microscope. **b**, Five regions for the fabricated sample analyzed by the microscope and compared to the designed mask.

6. Radiative coupling to the continuum due to the inversion symmetry breaking

The eigenmodes are numerically calculated using the COMSOL Multiphysics software, as shown in **Figure S8**, where both devices, i.e., lattice constant of $a = 31$ μm and a $= 30$ μm, were calculated. Initially, the passive MZM cavity is treated as the perfect electric conductor (PEC) cladded PhC, and the drilled air cylinder holes are perfectly matched with the given parameters (**Figure 2** in main text). For the sample with $a = 31$ μm, a mid-gap state at 3.19 THz (~3.26 THz for sample $a = 30$ μm), i.e., the photonic analogue of Majorana zero mode bound to a vortex, is clearly observed between two

doubly degenerated band edges (at 3.05 THz (~3.11 THz for sample $a = 30$ μm) and 3.38 THz (~3.45 THz for sample $a = 30$ μm), respectively) opened by the intervalley coupling discussed above. Then the linear expansion of the air cylinder holes is taken into consideration. The metallic claddings are gold film with thickness of 600 nm that is depicted by the Drude model. In addition, the radiuses of dilled air cylinder holes in the QCL slab have a 300-nm expansion compared to the drilled air cylinder holes in the top Au electrode contact. Finally, the air cylinder holes are all treated as elliptical shapes with ratio of long axis divided by short axis as 1.05, and major axis along y-axis, as shown in **Figure S7**. The lower and upper band edges (LBEs and UBEs) show splitting due to the elliptical air holes in the realistic device introduce anisotropy, which also increase the radiative losses to decrease the Q-factor of the MZM cavity. All the modes are now visualized at around 3.16 THz for the LBEs (~3.32 THz for sample $a = 30$ μm), 3.34 THz for the MZM mode (~3.52 THz for sample $a = 30$ μm), and around 3.52 THz for the UBEs (~3.73 THz for sample $a = 30$ μm). To evaluate the lasing capabilities of both BE states and mid-gap MZM state, the Purcell factors were calculated. As finite size of the lattice is investigated, the Purcell factor is equivalent to the local density of state that take care two key parameters of a cavity: quality factor Q and modal volume V [4]. In a realistic system, the total Q-factor is determined by three types of loss mechanisms, the intrinsic radiation loss $1/Q_{rad}$, the material absorption loss $1/Q_{abs}$, and the radiative loss due to surface roughness $1/Q_{sur}$. Therefore, the total loss rate reads $1/Q_{tot} = 1/Q_{rad} + 1/Q_{abs} + 1/Q_{sur}$. By using acid to remove the residuals on the surface of our sample, we've already minimized the scattering loss due to surface roughness, as shown in **Figure S6**. The mode volume, defined as $V = \frac{\int \varepsilon |E|^2 dV}{max(\varepsilon |E|^2)}$ with ε as the dielectric permittivity of QCL, is a value to measure the volume within which the mode is confined. The Purcell factor is therefore written as

$$F = \frac{3Q}{4\pi^2 V} \left(\frac{\lambda}{n_{eff}} \right)^3. \tag{S2}$$

Here λ is the vacuum wavelength and $n_{eff} = \sqrt{\frac{\int \varepsilon |E|^2 dV}{max(|E|^2)}}$ is the effective index of refraction. Therefore, even though the Q-factor of the MZM state seems not as high as these sitting at the UBE, its bounded

feature to the vortex core indicates the smallest mode volume and the highest Purcell factor, as shown in **Figure S8**. These have been proved to fit very well with the experimentally measured laser spectra in the main text.

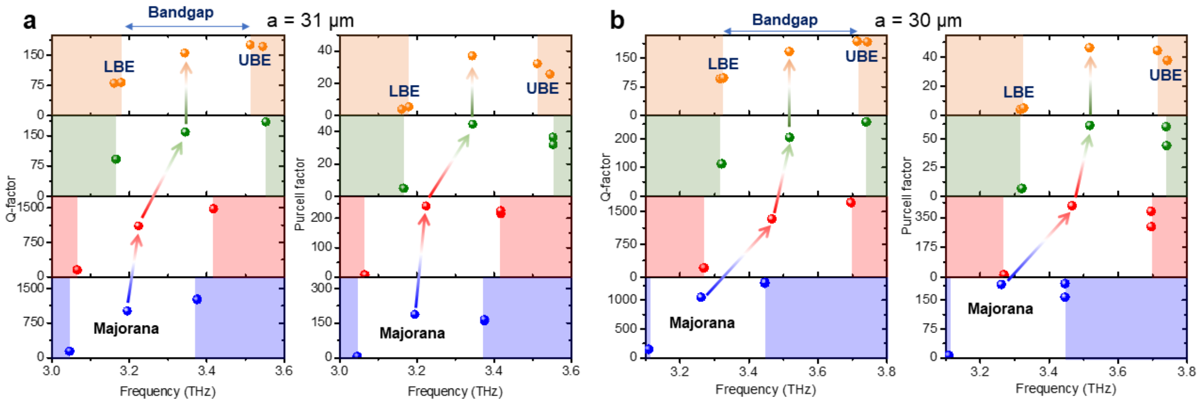

Figure S8. Calculated *Q*-factor and Purcell factor of the device. From bottom to top: PEC cladded MZM cavity without expansion, PEC cladded MZM cavity with expansion, Au (Drude model) cladded MZM cavity with expansion and $r_{QCL} - r_{Au} = 400$ nm, Au (Drude model) cladded MZM cavity with expansion, anisotropy (semi-major axis by semi-minor axis equals to 1.05, and orientation along *y*-axis).

Even though the lattice is hexagonally distributed and the intervalley phase is smoothly interpolated, the discrete and nonperiodic features of the spatially arranged air cylinder holes imply that the system sustains radiative coupling to the radiation continuum due to the inversion symmetry breaking of the intervalley phase. It clearly from the top panel of **Figure S9** that the cavity still tightly confines the field even there is anisotropy present in the system and lossy materials are taken into consideration. The Fourier transformed fields $FT\{E_z\}$ are plotted to show that the MZM mode indeed exists within the light cone, as shown in the middle panel of **Figures S9**. In addition, the radiation capability increases form **Figure S9a** to **S9d** as the center portion is getting stronger even air hole size expansion and anisotropy are taken into numerical calculations. Moreover, compared to the cavities with circular air cylinders, the device with elliptical air cylinders shows asymmetrical Fourier transformed fields $FT\{E_z\}$, as shown by the middle panel of **Figure S9d**. Therefore, the radiative far field also presents asymmetric beam profile. Overall, all the far-field beams present doughnut-shaped beam profiles, and the vector electric field (black arrows) indicates that the CV beam under

investigation is spiral polarization. The corresponding 3D simulation of the whole QCL device is shown in **Figure S10**. The emitted far-field beam profile is collected in a half-spherical space (perspective view), as shown in **Figure S10a**, and the 2D view at xz-plane is shown in **Figure S10b**, based on which the divergence angle is calculated.

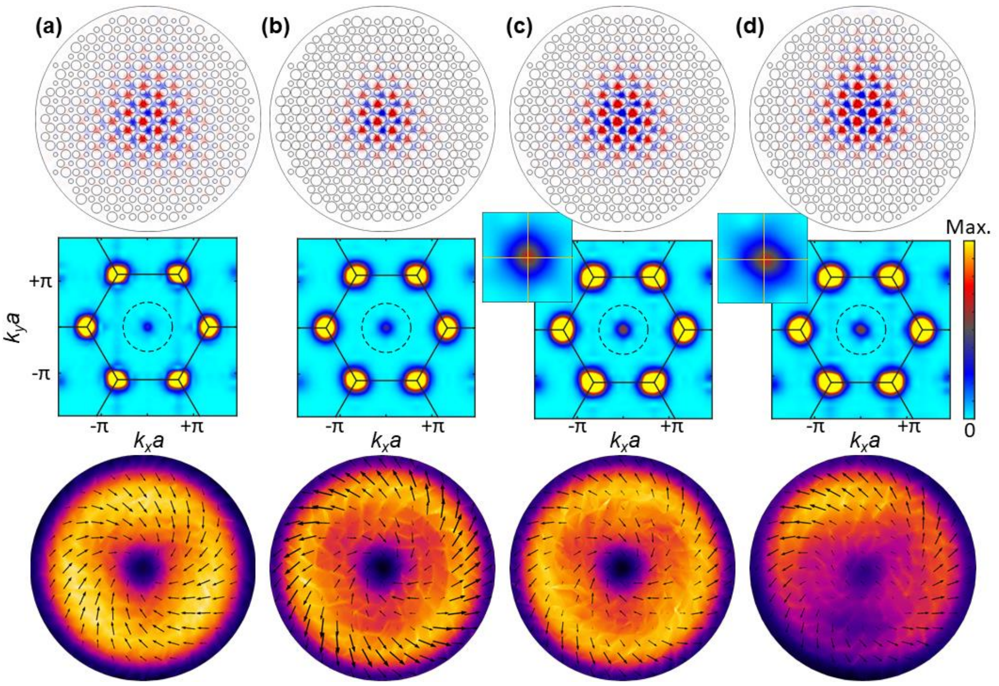

Figure S9. Top panel: electric field z-component at the middle of the MZM cavity. Middle panel: Fourier transformed electric field $FT(E_z)$. Bottom panel: Far-field beam patterns.

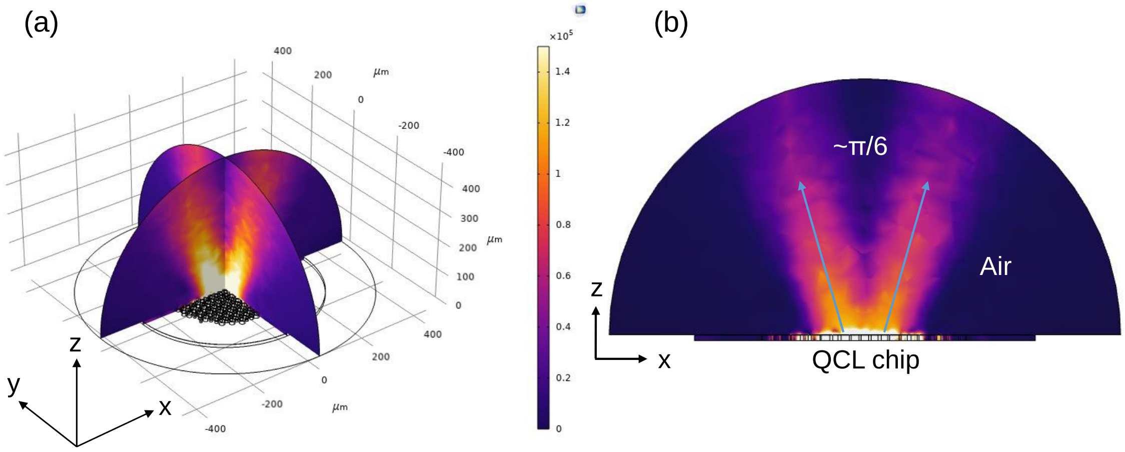

Figure S10. Perspective 3D view of the emitted laser beam profiles and the corresponding 2D planar view at y = 0 plane.

7. Defect presentence of photonic MZM laser

To investigate the topological robustness of our laser device, we fabricated THz QCL samples with winding number of $w = \pm 1$, and pumped the samples at their highest gain. Then the pulse duration is modulated from 100 ns to 800 ns that correspond duty cycles of 0.1% to 0.8% as the repetition rate is set as 10 kHz, as shown in **Figures S11a** and **S11b**. The stable lasing with single peak requests that the pulse width is larger than 200 ns for both sample with different winding numbers. However, a random defect with size around one period is accidentally generated in the intervalley coupling (phase control) domain for the sample with minus winding number, as shown in **Figure S11b**. Even so, the $w = -1$ sample also shows robust lasing at same frequency that is around 3.51 THz as the $w = +1$ sample. In the passive system [3], this topological robustness has been discussed by employing symmetry-preserved defects in the vortex core, but here, we prove that the topological robustness is still preserved when there is a randomly generated defect in the intervalley coupling domain without any symmetric characteristics.

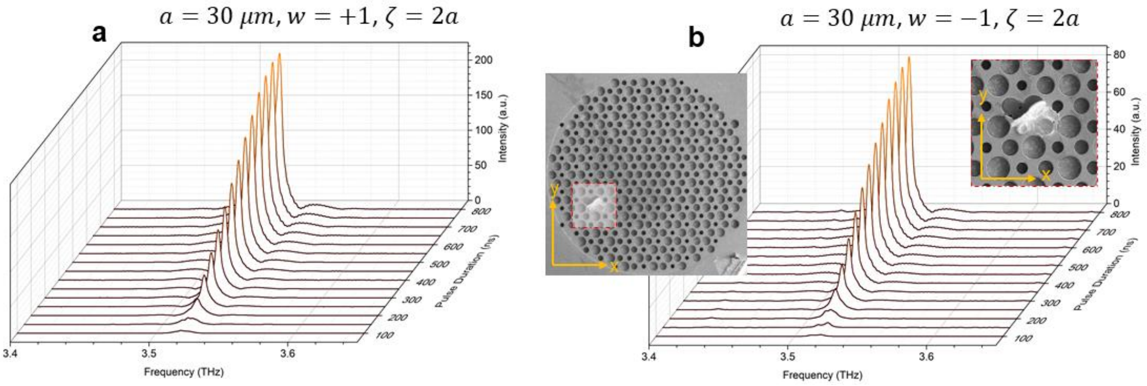

Figure S11. Robustness of the single-mode QCL at different pulse durations. **a**, winding number +1 without defect and **b**, winding number -1 with accidental defect due to fabrications.

8. Polarization-resolved lasing spectra

Before mapping the far-field beam pattern, the polarization-resolved lasing spectra were characterized, as shown in **Figure S12**. It is clearly observed that the photonic MZM laser peak robustly dominates the emission spectrum. Once again, the slight variation of the intensity is due to the anisotropy of fabrication.

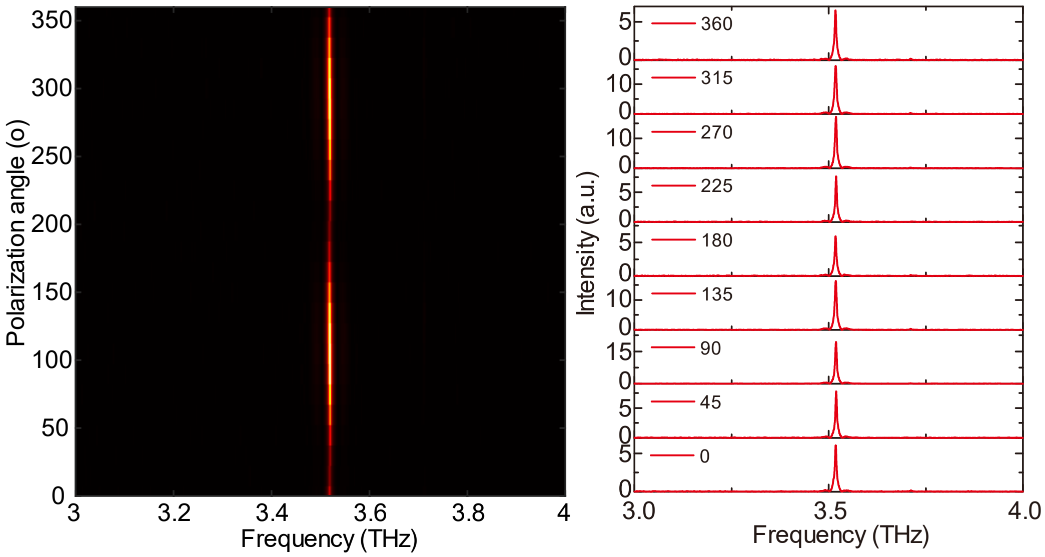

Figure S12. Polarization-resolved lasing spectra for sample with lattice constant of *a* = 30 μm.

9. Sample alignment for far-field mapping

The physics of light diffraction has substantial importance for fundamental sciences and industry applications. Here, we employ the Littrow configuration to align the QCL, focal lens, and Golay cell detector. When a light with wavelength of λ is incident on the MZM topological cavity with angle of θ_i to the normal, the diffracted beams are observed at angle of θ_d, thus $sin(\theta_i) - sin(\theta_d) = m\frac{\lambda}{a}$, where *a* is the lattice constant, and *m* is the order of diffraction. If θ_i = -θ_d, we have $sin(\theta_d) = m\frac{\lambda}{2a}$. The diffracted beam corresponding to order m then exactly retraces the incident beam. The zero-order diffraction *m* = 0, corresponding to specular reflection ($\theta_d = 0$), is employed to get our experimental setup aligned. As shown in **Figure S13a**, a laser point fixed on the mechanical arms was incident on the QCL, and the diffracted beams show hexagonal patterns. However, the zero-order diffraction shows maximum intensity at the center. Therefore, by setting an iris between the laser pointer and QCL (inside cryostat chamber), the zero-order diffraction must be a specular reflection and exactly projects the maximum intensity to the hole of the iris so that the THz QCL is aligned, as shown in **Figure S13b** and **S13c**. Then a focal lens was also inserted between the iris and the THz QCL sample, as shown in **Figure S13d**. The laser pointer gets focused and projected on the same position of THz QCL sample. Finally, the laser pointer was replaced by a THz Golay cell detector, as shown in **Figure S13e**.

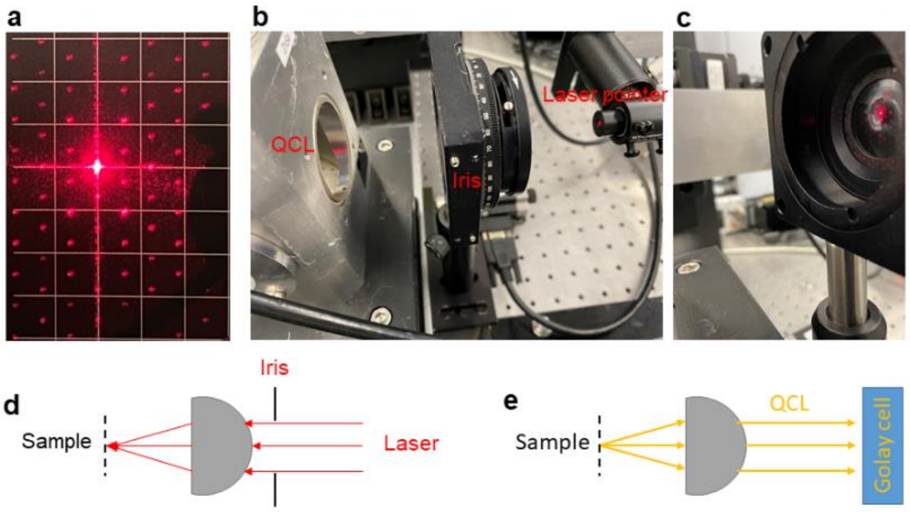

Figure S13. **a**, Diffracted laser beams imaged on a screen showing hexagonal patterns. **b**, **c**, A simple alignment system that requires the zero-odder diffraction to be specular reflection and the diffraction center reverses back to the hole of iris. **d**, A focal lens is inserted between the THz QCL sample and iris, where the focused light should be on same position of the THz QCL sample. **e**, Electrically pumped the QCL with the laser pointer replaced by a THz Golay cell detector for far-field mapping.

10. Power measurement for the photonic MZM laser

The QCL power was measured by a terahertz powermeter, Gentec-EO T-Rad with detector head of THZ9B-BL-DZ-D0. The THz detector has a collection aperture of 0.9 mm in diameter. To improve the collection efficiency, the laser emission was focused by two parabolic mirrors, as shown in **Figure S14**. Sample with lattice constant of a=30 μm, winding number w=-1, the vortex core of 8*a, and width of Kekule modulation region is 9*a, was measured. The pump pulse width was 500 ns and repetition rate were 10 kHz, which were also used for far-field measurement. In a dry nitrogen purging circumstance, the measured average powers are 22.6 μW for sample with a=30 μm and vortex core of 8*a. The effective pumping area (0.103 mm^2) is slightly smaller than a ridge laser (ridge width 100 μm and ridge length 1500 μm for total area of 0.15 mm^2), but the laser delivers a peak power > 9 mW that is much higher than the ridge laser with a peak power of ~1.54 mW. The power efficiency of the THz vector beam laser is improved by around 9-fold.

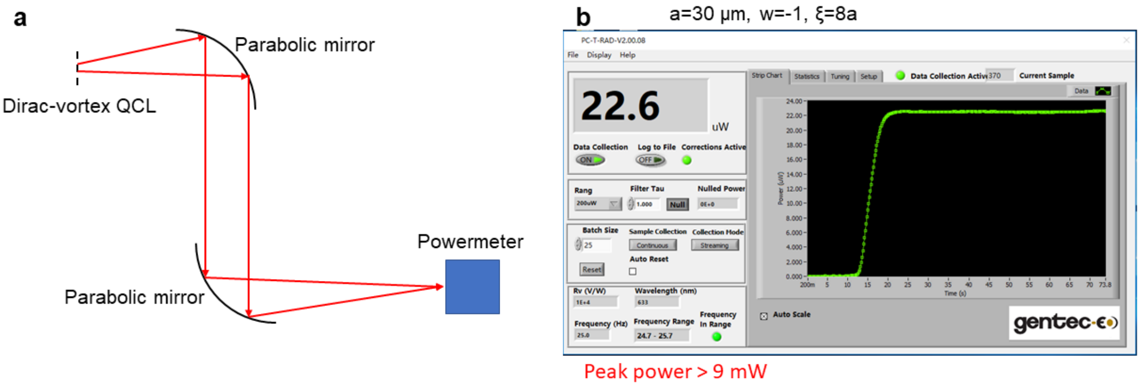

Figure S14. **a**, A simple setup for focusing the diverged beam of the photonic MZM QCLs. **b**, The captured power measurement software screenshot for the QCL device.

References

1. Vitiello, M. S. & Tredicucci, A. Tunable emission in THz quantum cascade lasers. IEEE Trans. Terahertz Sci. Technol. 1, 76-84 (2011).
2. Fathololoumi, S. et al. Terahertz quantum cascade lasers operating up to ~200 K with optimized oscillator strength and improved injection tunneling. Opt. Express 20, 3866-3876 (2012).
3. Gao, P. et al. Majorana-like zero modes in Kekulé distorted sonic lattices. Phys. Rev. Lett. 123, 196601 (2019).
4. Liang, X. & Johnson, S. G. Formulation for scalable optimization of microcavities via the frequency-averaged local density of states. Opt. Express 21, 30812-30841 (2013).
