# Supplementary material for: Photonic Majorana quantum cascade laser with polarization-winding emission
Source: Nat Commun. 2023 Feb 9;14:707. doi: 10.1038/s41467-023-36418-y (PMC9911720; doi:10.1038/s41467-023-36418-y)
Supplement: Supplementary file 3 — Lasing Reporting Summary [file 41467_2023_36418_MOESM3_ESM.pdf]

nature research

Corresponding author(s): Qi Jie Wang (Prof.): qjwang@ntu.edu.sg

Lasing Reporting Summary

Nature Research wishes to improve the reproducibility of the work that we publish. This form is intended for publication with all accepted papers reporting claims of lasing and provides structure for consistency and transparency in reporting. Some list items might not apply to an individual manuscript, but all fields must be completed for clarity.

For further information on Nature Research policies, including our data availability policy, see Authors & Referees.

Experimental design

Please check: are the following details reported in the manuscript?

1. Threshold

Plots of device output power versus pump power over a wide range of values indicating a clear threshold

☒ Yes ☐ No

Figure 3d and Figure 5a show the L-I-V curve and the corresponding laser thresholds.

2. Linewidth narrowing

Plots of spectral power density for the emission at pump powers below, around, and above the lasing threshold, indicating a clear linewidth narrowing at threshold

☐ Yes ☒ No

The spectral linewidth of emission device is ~ 0.16511 cm-1 (Figure 3d), which is comparable to that of a conventional ridge laser (Figure S1a). It also shows a sharp power increase above a certain pump current density (laser threshold) with its maximum power also comparable to that of the ridge laser. Quantum cascade laser is well developed. This is a typical feature of QCL lasing, which is widely studied and accepted. Thus, it is not necessary to further characterize the linewidth narrowing process.

Resolution of the spectrometer used to make spectral measurements

☒ Yes ☐ No

The spectra were captured by a Fourier transform infrared spectrometer (FTIR, Bruker Vertex 80 series) with a room-temperature deuterated triglycine sulfate (DTGS) detector. The spectral resolution is 0.08 cm-1.

3. Coherent emission

Measurements of the coherence and/or polarization of the emission

☒ Yes ☐ No

The polarization measurement results are presented in Figure 4. The farfield beams with/without polarizer are well matched with the numerical calculations.

4. Beam spatial profile

Image and/or measurement of the spatial shape and profile of the emission, showing a well-defined beam above threshold

☒ Yes ☐ No

Figure 4 plots the far-field beam profile of the proposed QCL above it's threshold. The far-field beam shows doughnut-like pattern that is a demonstration of cylindrical vector (CV) beam. The beam profile was also double confirmed by putting a linear polarizer at the emission side of the laser. By rotating the polarizer, two lobes of the far-field beam were captured. This confirms the CV beam and it's spiral polarization.

5. Operating conditions

Description of the laser and pumping conditions
Continuous-wave, pulsed, temperature of operation

☒ Yes ☐ No

The pump condition is described in the "Method->Characterization" section.

Threshold values provided as density values (e.g. W cm^{-2} or J cm^{-2}) taking into account the area of the device

☒ Yes ☐ No

Figure 3d and Figure 5a show the QCL threshold is around 1.2 kA/cm^2 taking into account the real device pumping area.

6. Alternative explanations

Reasoning as to why alternative explanations have been ruled out as responsible for the emission characteristics
e.g. amplified spontaneous, directional scattering; modification of fluorescence spectrum by the cavity

☐ Yes ☒ No

The L-I-V curve characteristics (clear threshold and roll-over points), high power (~9 mW in peak power), narrow linewidth, clear and pure farfield beam well matched with theoretical calculation, all these clues together exclude the alternative explanations of emission mechanism in terahertz quantum cascade gain medium. All the observed lasing features are typical for well-studied quantum cascade laser. This work presents a laser with special far-field pattern which is not observed in typical (e.g. ridge) quantum cascade lasers.

7. Theoretical analysis

Theoretical analysis that ensures that the experimental values measured are realistic and reasonable
e.g. laser threshold, linewidth, cavity gain-loss, efficiency

☒ Yes ☐ No

Figure 3c shows the cavity gain-loss competition that stems from the numerical calculated cavity loss (linewidth) and theoretically calculated gain curve of the QCL wafer.

8. Statistics

March 2018

Number of devices fabricated and tested	☒ Yes<br>☐ No	Two groups of samples were fabricated and measured. Figures 3 and S10 are the comparison between them.
Statistical analysis of the device performance and lifetime (time to failure)	☒ Yes<br>☐ No	Semiconductor lasers are always robust. Therefore, the laser devices are usually pumped for more than 48 hours for mapping out the far-field beam profiles, after which the laser still works well.
